# Subacute Thyroiditis—Is it Really Linked to Viral Infection?

**DOI:** 10.1210/clinem/dgaf023

**Published:** 2025-01-15

**Authors:** Hans Martin Orth, Alexander Killer, Smaranda Gliga, Michael Böhm, Torsten Feldt, Björn-Erik O Jensen, Tom Luedde, Rolf Kaiser, Martin Pirkl

**Affiliations:** Department of Gastroenterology, Hepatology and Infectious Diseases, Medical Faculty and University Hospital Düsseldorf, Heinrich-Heine-University Düsseldorf, 40225 Düsseldorf, Germany; Department of Gastroenterology, Hepatology and Infectious Diseases, Medical Faculty and University Hospital Düsseldorf, Heinrich-Heine-University Düsseldorf, 40225 Düsseldorf, Germany; Department of Gastroenterology, Hepatology and Infectious Diseases, Medical Faculty and University Hospital Düsseldorf, Heinrich-Heine-University Düsseldorf, 40225 Düsseldorf, Germany; Institute of Virology, Faculty of Medicine and University Hospital Cologne, University of Cologne, 50937 Cologne, Germany; German Center for Infection Research (DZIF), Partner Site Cologne-Bonn, 50937 Cologne, Germany; Department of Gastroenterology, Hepatology and Infectious Diseases, Medical Faculty and University Hospital Düsseldorf, Heinrich-Heine-University Düsseldorf, 40225 Düsseldorf, Germany; Department of Gastroenterology, Hepatology and Infectious Diseases, Medical Faculty and University Hospital Düsseldorf, Heinrich-Heine-University Düsseldorf, 40225 Düsseldorf, Germany; Department of Gastroenterology, Hepatology and Infectious Diseases, Medical Faculty and University Hospital Düsseldorf, Heinrich-Heine-University Düsseldorf, 40225 Düsseldorf, Germany; Institute of Virology, Faculty of Medicine and University Hospital Cologne, University of Cologne, 50937 Cologne, Germany; German Center for Infection Research (DZIF), Partner Site Cologne-Bonn, 50937 Cologne, Germany; Institute of Virology, Faculty of Medicine and University Hospital Cologne, University of Cologne, 50937 Cologne, Germany; German Center for Infection Research (DZIF), Partner Site Cologne-Bonn, 50937 Cologne, Germany

**Keywords:** subacute thyroiditis, respiratory virus, SARS-CoV-2, echovirus, coxsackievirus

## Abstract

**Context:**

Subacute thyroiditis (SAT) is a painful inflammatory disorder of the thyroid gland, which—after a phase of thyrotoxicosis—leads to transient, or less frequently permanent hypothyroidism. Apart from a strong association with specific human leukocyte antigen alleles, the causes are uncertain. Viral disease has been hypothesized as a trigger, with enteroviruses, namely echovirus and coxsackievirus, showing a seasonal distribution that coincides with the incidence of SAT.

**Objective:**

In the first year of the COVID-19 pandemic, strict hygiene measures led to a sharp decline in infections and thus offered the opportunity to test this hypothesis.

**Methods:**

We analyzed national registry data of hospitalized patients from Germany during the years 2015 to 2022 (Federal Statistical Office [Destatis], Wiesbaden, Germany) and surveillance data on infectious diseases from the same years (clinical-virology.net and RKI). Statistical analysis includes modeling of seasonality by month, polynomial autoregression, and Granger causality to assess dependency of future SAT frequencies from past ones, and association of virus incidence to SAT frequency, respectively.

**Results:**

Our study confirms previously described epidemiological findings with higher incidence in women and a seasonal peak in late summer coinciding with the seasonality of enteroviruses until 2019. In 2020, the pattern remained unchanged, except for the marked reduction of enteroviruses and other pathogens (except SARS-CoV-2) due to hygienic measures. Moreover, the SAT seasonality in the years 2021 and 2022 was apparently unaltered through the COVID-19 pandemic.

**Conclusion:**

Our study provides strong evidence that despite their seasonal pattern, Echoviruses and Coxsackieviruses are not the cause of SAT. Moreover, no other analyzed virus (including Influenza A and B, Parainfluenza, Rhinovirus, Human Coronaviruses including SARS-CoV-2) showed any association.

Subacute thyroiditis (SAT) or—after its first describer—de Quervain thyroiditis is one of the less frequent causes of thyroid disease. A community study from 2003 calculated an incidence of 4.9 per 100 000/year (1). The disease is characterized by a migratory, painful swelling of the thyroid gland, sometimes accompanied by fever, malaise, and night sweats. In a retrospective cohort study including 852 patients, only 28.2% of the included patients experienced fever exceeding 38 °C. In the same study, 23% of patients had reported respiratory symptoms preceding the diagnosis of SAT (2). Dry cough, however, may rarely occur during the acute phase of SAT, most likely due to tracheal compression (3, 4). Following the initial phase, a transient thyrotoxicosis develops due to release of thyroid hormones from disrupted follicle cells. Symptoms usually subside after several weeks, followed by a phase of hypothyroidism. Full recovery is observed in about 3 out of 4 patients. During the acute phase, treatment with nonsteroidal anti-inflammatory drugs and systemic corticosteroids is often used to alleviate symptoms but may not have influence on later development of permanent hypothyroidism (5).

Causes and risk factors for SAT are only partly understood. Besides a strong association with HLA-B*35, other human leukocyte antigen (HLA) alleles have been associated with an increased incidence, as well (6). As in many other autoimmune disorders, females are affected more often than males. No specific autoantibody profiles have conclusively been linked with subacute thyroiditis, although transient positivity for antithyroglobulin and antithyroid peroxidase antibodies has been observed in some patients (7). Early reports suggested a connection with viral infections due to similar symptoms (8). Various viral infections have been hypothesized as possible triggers for SAT, including Foamy virus, mumps virus, Epstein-Barr virus (EBV), cytomegalovirus (CMV), and other viruses. This is mostly based on detection of virus-like particles by electron microscopy and sporadic detection of viral nucleic acid patterns or virus cultivation in cell culture or xenodiagnosis. The majority of these publications date to the 1970s or earlier and include few cases. Moreover, significant changes in antibody titers against various viruses (eg, mumps virus) were pointed to for explaining recent infection (8, 9). Besides viral infection, there have been anecdotal reports of SAT occurring in temporal connection with vaccinations even before the COVID-19 pandemic (10).

Seasonality of SAT was first described by Saito et al in 1974 (11) with a peak during hot summer months. This was confirmed by 2 studies from Italy and Japan and led to the assumption that SAT may be associated with Enteroviruses, in particular echovirus and coxsackieviruses, which reach their peak circulation at the same time, although the larger Japanese study showed no statistical accumulation of infectious diseases in the patients affected (2, 12). A small study conducted to test this hypothesis did not detect Enterovirus RNA in tissue samples of patients with acute disease (13). In another small study targeting EBV and CMV, no viral DNA could be detected in fine-needle aspirations, either (14). Prospective studies to detect specific viral infections preceding SAT as a possible trigger for autoimmunity are almost impossible to conduct due to the low incidence of SAT. Beside viral infection of thyroid tissue, autoimmune response to viral infection in connection with specific HLA alleles is currently the most highly favored hypothesis for the etiology of SAT.

The SARS-CoV-2-pandemic offers a special opportunity for testing numerous hypotheses. Due to strict hygiene measures, especially in 2020—the first year of the pandemic—significantly fewer respiratory, gastrointestinal, and other infections (such as measles) occurred (15, 16), thus leading to the expectation of a lower incidence of causally associated immunological diseases. This situation has been used in a few studies, including single-center case series from Italy and Turkey, as well as a national registry analysis from South Korea (17-20). The results are ambiguous, and while the 3 single-center studies did not observe any difference in total case numbers of SAT in 2020 compared to previous years, the Korean study detected a small, but significant, increase from 7.27 to 8.30 per 100 000 individuals. The Korean study also mentions a decrease of related respiratory viral infections other than COVID-19.

During the COVID-19 pandemic, numerous cases of SAT have been published with temporal relation not only to SARS-CoV-2 infections but also to vaccinations. In some of these publications, the temporal connection was defined strictly for cases of SAT occurring simultaneously with COVID-19, while other publications chose a generous definition with an interval of up to several months between COVID-19 and SAT (21). In several reported cases, an atypical clinical pattern lacking neck pain is described (22), which however may be related to nonthyroidal illness syndrome in critically ill patients, or to thyroidal tissue damage due to infection of the thyroid gland with SARS-CoV-2 (21, 23). A recent study reported on 258 cases of SAT occurring within 3 months after SARS-CoV-2 vaccinations and found no clinical difference compared to 98 cases of SAT occurring within 3 months after COVID-19 or 455 control patients (24).

In this study, we analyze national registry data of hospitalized patients from Germany during the years 2015 to 2022, as well as surveillance data on respiratory and several other infections.

## Materials and Methods

### Data Acquisition

In Germany, the accounting of hospital treatments with the health insurance companies is based on International Classification of Diseases, Tenth Revision (ICD-10) codes. ICD-10-GM, a national adaptation of ICD-10-WHO, was first introduced in 2000 and is also used for statistical purposes and quality management (25). The data set of all inpatient treatments is available to the Federal Statistical Office (Destatis, Wiesbaden, Germany). Epidemiological data can be retrieved there on request. According to a contractual agreement between Destatis and the University Hospital Düsseldorf, we obtained data on all patients diagnosed with the ICD-10 code E06.1 (subacute thyroiditis) during the years 2015 to 2022. Other etiologies of thyroiditis such as Hashimoto thyroiditis, silent thyroiditis, lymphocytic thyroiditis, drug-induced thyroiditis, postpartum thyroiditis, or pyogenic thyroiditis have different ICD-10 codes and are therefore not included in this study.

Besides the total number of hospitalizations and average duration of in-hospital treatment, the figures available from Destatis include the number of hospitalized cases of SAT for each year, stratified by age, sex, and month of diagnosis. The data do not include individual diagnostic or clinical information. Since these data are provided in an anonymized fashion, ethical approval is not required.

The data of the clinical virology net aims to afford clinicians and scientists an overview on the current epidemiological spread of infectious diseases and pathogens. The project started in 2009 with the collection of test results on respiratory viruses and expanded over time to include respiratory viruses and bacteria and later also gastroenterological pathogens. Laboratories can decide to join the network and then provide anonymized data on test results. So far, more than 60 centers provide data, with an increasing number of test records being uploaded continuously. The data are mostly from university-based clinics but also from private laboratories mostly from Germany and Austria. The database comprises positive and negative results from patients tested for up to 18 different viruses (including influenza, respiratory syncytial virus [RSV], and SARS-CoV-2), 8 different bacteria, and additionally 5 viruses related to gastroenteritis. A plausibility check with the data from the Robert Koch Institute on influenza showed agreement between seasonality and frequency between the two databases. The Respiratory Virus Dashboard and the Gastro Dashboard are freely available online (https://public.clinical-virology.net/) (26).

Additional epidemiological data on CMV, hepatitis C and E, measles, and HIV were retrieved from SurvStat@RKI 2.0, an online platform for epidemiological data provided by the Robert-Koch-Institute, the German public health institute (27).

### Statistical Analysis

We used the statistical software R (R Core Team; 2023. R: A Language and Environment for Statistical Computing. R Foundation for Statistical Computing. https://www.R-project.org) to analyze the data. Seasonality of SAT frequency was modeled by fitting a model to the data with year (linear) and month (polynomial with degrees 0 to 6) as predictors. The final model used only months as the predictor and thus the prediction is the same for every year. The prediction is not a smooth interpolation because our model predicts each individual month and not fractions like month 1.5.

We analyzed the predictive power of the time series by training a polynomial model (polynomial autoregression) with previous time points as predictors for the current time point. We used 10-fold cross-validation to optimize the hyperparameters *polynomial degree* (1, 2, 3, 4, 5) and *lag* (1, 2, 3, 4, 5), the number of previous time points to include in the model. Due to longitudinal dependency, folds cannot be constructed randomly. Hence, we randomly sampled a time point as the start with the *n* next time points as the training set and the *n* time points following those as the test set. We repeated this 10 times. We assessed model fit during cross-validation with mean squared error (MSE) as the loss function.

We associated the SAT samples with other viruses by computing pairwise correlations. We assigned *P* values to correlation coefficients applying Fisher transformation (28, 29). We adjusted for multiple testing with the false discovery rate (Benjamini-Hochberg). In a stepwise forward regression we ranked the pathogens by how significantly their additional inclusion improves fit compared to the model not including the pathogens. Model fit improvement was assessed by a likelihood ratio test (R package “lmtest’) (30). Granger causality (31) adds a covariate to autoregression if this improves predictiveness of the model. We test this predictive power with the function “grangertest’ (R package “lmtest’), which uses a Wald test (32, 33) for significance computation of the coefficient of the covariate. We modeled the underlying trend of SAT frequency independently for each year by locally weighted polynomial regression (R function “lowess”).

## Results

**Figure 1.**
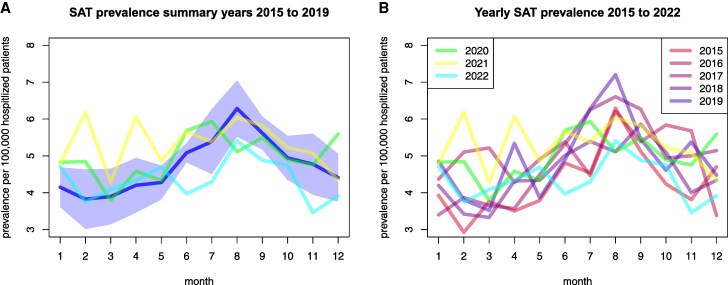
The mean over the years 2015 to 2019 with SD overlaid by the years A, 2020, 2021, and 2022 and B, individual curves of all years.

According to the Destatis data, 6856 cases of SAT were diagnosed in hospitalized patients during the study period from 2015 to 2022. The majority (n = 4,908, 72%) were female. In all 8 years, the vast majority of cases occurred in the fifth and sixth decade of life, with the mean age at diagnosis ranging from 51.00 years in 2015 to 52.74 years in 2021. Average hospital stay was 5.25 days. The average number of SAT diagnoses was 891 during the prepandemic years 2015 to 2019; the total number in 2020 was lower (n = 829), but in 2020 also the total number of hospital admissions (n = 16 359 312) was lower than the average of 2015 to 2019 (n = 18 821 667). Therefore, the frequency of SAT per 100 000 hospital admissions in 2020 was slightly, yet not significantly, higher than the average from 2015 to 2019 (5.07 vs 4.73 ± 0.29). The total number of SAT cases was 863 in 2021 and 709 in 2022, respectively. The incidence per 100 000 hospitalized patients was highest in 2021 with 5.31 and lowest in 2022 with 4.36. Fig. 1 displays the monthly distribution of SAT during the study period. The summary shows a clear seasonality (Fig. 1A), but individual years can have very dissimilar curves (Fig. 1B). The overall trend after 2020 is similar to the years before SARS-CoV-2 except for the year 2021 with 2 peaks (outliers) in winter/spring (see Fig. 1B). We computed the underlying theoretical distribution in the data by fitting a lowess regression, independently for each year (2015-2022; Supplementary Fig. S1 (34)). All models show the theoretical peak in August, except for 2016, where the peak is in September. Seasonality is similar over all years with some variance in the vertical shift.

### Seasonality of Subacute Thyroiditis

**Figure 2.**
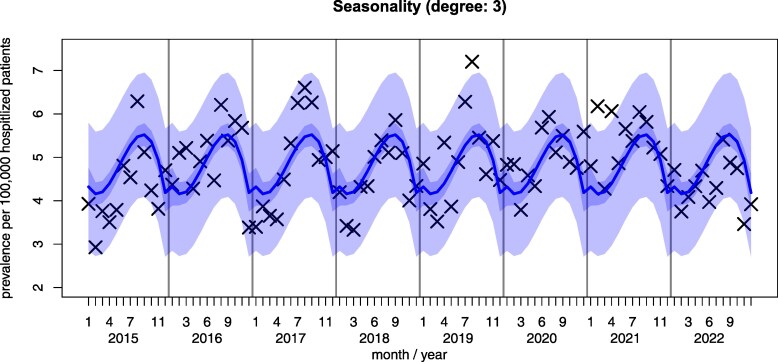
Seasonality of subacute thyroiditis prevalence. Small and large intervals around the fitting curve show CI and prediction interval, respectively. The model was learned from the 12 months with each year (2015-2022) as a sample replicate. The model is the same for each year, and here we visualize the individual annual model fit to the data. The roughness of the model curve is because we only predict discrete values from 1 to 12.

Seasonality was optimally modeled by a polynomial of degree 3 (Fig. 2). Lower degrees suffered from bad fit (adjusted *R*^2^, Supplementary Fig. S2H (34)) to the data, and higher degrees added little to the overall model fit (Supplementary Fig. S2A-S2C, S2E-S2G (34)). Naturally, our model confirmed the covariate months as most valuable for modeling seasonality with June to October as most significant and with effects peaking in August. We observed a small positive effect for covariate year, but due to low effect size and a not statistically significant *P* value (>.05), we removed it from the final model (Fig. 2). This shows the predicted seasonality for the variable month as the predictor with raw data depicted as Xs. That is, using only the current month, yearly seasonal trends of SAT frequency can already provide a reasonable estimate.

**Figure 3.**
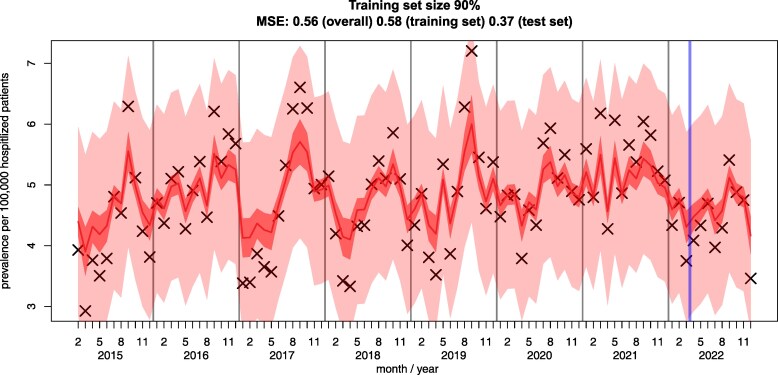
Predictability from previous time points. Small and large intervals around the fitting curve show CI and prediction interval, respectively. Xs show the raw data. The thick vertical line between March and April 2022 indicates the transition from training set to test set. The thin vertical lines indicate transition between years.

In the next analysis we assessed how well a model learned by polynomial autoregression generalizes, that is, how well do previous time points of SAT-positive rates predict the current rate. We used 10-fold cross-validation to optimize hyperparameters *lag* (the number of previous time points to include) and *polynomial degree*. We used 90% of the data (2015-mid 2022) as the training set and sampled 10 folds. We repeated this 10 times. The optimal model had degree 1 and lag 4; however, its MSE was not statistically smaller than the MSE of the most conservative model with degree 1 and lag 1, that is, we predict the next week based on the current week with a linear function. The model shows reasonable generalization on the test set (Fig. 3). This shows the CI and prediction interval for the prediction (central line) and the raw data as Xs. To observe the effect the training set size has on the predictiveness, we set the training size for the final model to 25%, 50%, and 75% as well and predicted the test set (Supplementary Fig. S3A-S3C (34)). At a training set size of 25%, the model already generalizes well with a MSE lower than for the training set. This confirms that seasonality is highly characteristic for SAT independent of the year.

### Subacute Thyroiditis Association With Pathogen Infection Rates

**Figure 4.**
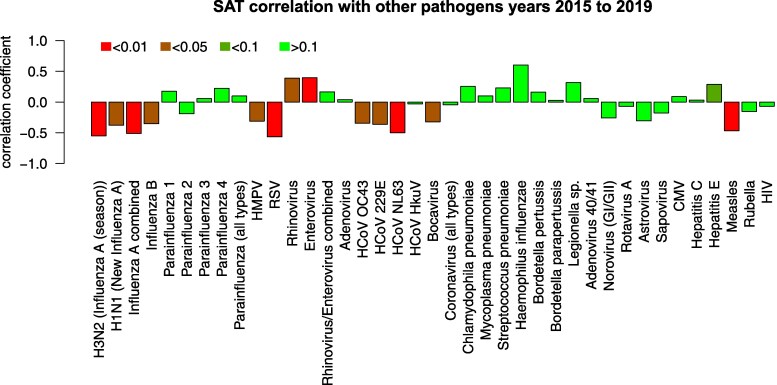
Correlation to other pathogens. Color shades show the significance of the correlation coefficient by way of Fisher transformation. Large but not statistically significant coefficients are due to a low number of samples.

We extended the analysis by analyzing associations with rates of infections by other pathogens in the time frame 2015 to 2019, because 2020 was heavily influenced by the COVID-19 pandemic. This was confirmed by a statistically significant (*P* < .05) negative correlation between almost all pathogens and the indicator variable—zero for years 2015 to 2019 and 1 for year 2020—that is, infection rates were virtually 0 or very low for most pathogens (Supplementary Fig. S4 (34)). Some pathogens exhibited a contrasting trend, namely human coronavirus (HCoV) HkuV, *Streptococcus pneumoniae*, *Haemophilus influenzae*, *Legionella*, *Moraxella catarrhalis*, *Cytomegalovirus*, and *Hepatitis E* (HEV). We started with an analysis correlating SAT frequency to pathogen infection rate (Fig. 4).

Several influenza viruses show a significant negative correlation to SAT frequency with a large correlation coefficient. Human metapneumovirus, RSV, several HCoVs, and bocavirus also show a significant negative correlation with relatively large correlation coefficients. Positively correlated with a significant coefficient are Enterovirus, Rhinovirus, and *Haemophilus influenzae*.

**Figure 5.**
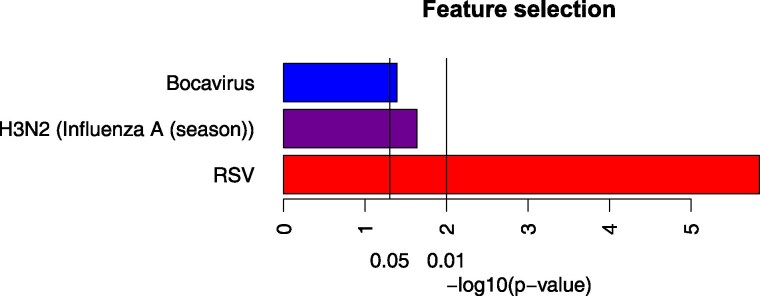
Feature selection. Pathogens, whose infection rates most significantly improve model fit for subacute thyroiditis frequency prediction. Vertical lines indicate statistical significance of .05 and .01, respectively.

We analyzed the possible predictive power of the different pathogens by a feature selection analysis using stepwise forward regression, that is, we started with a model including only the intercept. We added the pathogen with the largest improvement in model fit (likelihood ratio test). The new model includes intercept and the most significant virus. We repeated this until no pathogen significantly improved model fit. This feature selection removed many redundant viruses, like correlated influenza viruses and ranks RSV, H3N2, and bocavirus as significantly improving model fit (Fig. 5). The -log10(*P*-value) shows that RSV is deemed most important, followed by H3N2 and bocavirus.

**Figure 6.**
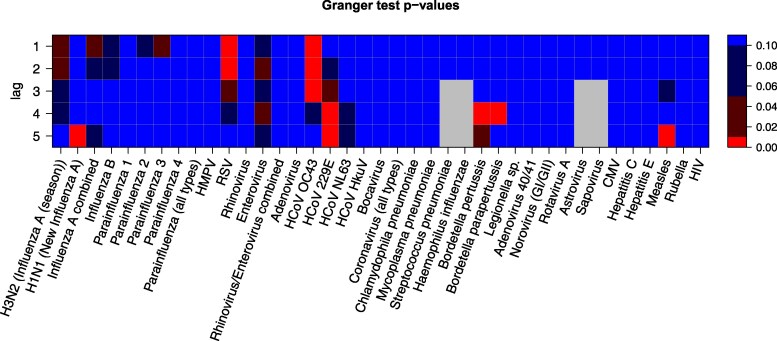
Granger test. *P* values from the Granger test over lag values 1 to 5 and all available pathogen infection rates. Different shades indicate different levels of statistical significance (.01, .05, .1, >.1).

Next, we investigated whether other viruses can improve the prediction of the current SAT frequency by including pathogen infection rates of previous months in the model, that is, we computed the model using only frequencies of SAT from previous months and compared this model with the model that additionally includes infection rates from previous weeks of another pathogen. The result of the Granger tests was partially similar to the correlations (Fig. 6). This figure shows the significance of each virus predicting future SAT frequencies based on lag, the number of weeks in advance. Influenza, RSV, enterovirus, and most HCoVs show a significant Granger causality for one or more lag values. However, we also observed that a significant correlation does not necessarily imply Granger causality. Rhinovirus, for example, shows no significance for any lag. On the other hand, parainfluenza 3 and *Bordetella (para-)pertussis* show significance for Granger causality without any significance in correlation.

### Subacute Thyroiditis and the SARS-CoV-2 Pandemic

We analyzed the frequency of SAT during 2020, the first year of the SARS-CoV-2 pandemic. We confirmed what is already observable in Fig. 1. The year 2020 showed basically no statistically significant difference in SAT frequency compared to previous years. However, pathogen infection rates generally dropped. Correlations of SAT frequency with pathogen infection rates for the year 2020 show partially large correlation coefficients (see Supplementary Fig. S3 (34)), but none are statistically significant due to the small sample size (12 months). From 2015 to 2019, the enterovirus infection rate was significantly positively correlated to SAT frequency, but during the year 2020 the correlation was not significant and was negative. Low enterovirus infection rates at the beginning of 2020 dropped to practically 0 for the rest of the year 2020 (Supplementary Fig. S5 (34)).

We repeated the analysis of 2020 with the years 2021 and 2022, and we also repeated the correlation analysis with other pathogens (Supplementary Fig. S6 (34)). We observed a statistically significant negative correlation with SARS-CoV-2 (*P *< .01) and a significantly positive correlation with *Haemophilus influenzae* (*P* < .05). We looked at both pathogens in comparison to SAT in more detail (Supplementary Fig. S7 (34)). The correlation to SARS-CoV-2 is mostly due to its countercyclic seasonality with high numbers in winter/spring and low numbers during summer, when SAT is at its peak. The correlation to *Haemophilus influenzae* follows the same logic, possibly related to rising SARS-CoV-2 numbers diminishing *Haemophilus influenzae* cases.

## Discussion

Molecular mimicry is one of the most frequently hypothesized concepts for development of autoimmunity. According to this theory, epitopes of infectious agents or their complexes with HLA molecules induce immunity against similar endogenous molecular structures of the host. For some autoimmune disorders and related infections, for example, Guillain-Barré syndrome and infection with *Campylobacter*, there is robust epidemiological evidence to assume causality (35). Likewise, a statistically significant association has been reported between several autoimmune conditions and specific HLA alleles, for example, HLA-B*27 and ankylosing spondylitis (36). The strong association of HLA-B*35 and SAT thus suggests a similar mechanism (6). It is therefore plausible that SAT and certain infectious agents may have a causal relationship. The previously observed seasonal correlation between SAT and enterovirus fits this theory well (2, 12). Our statistical analysis of 6856 SAT cases from the Destatis data confirms the seasonal pattern observed in the previous studies (n = 80 patients in the Italian study and n = 852 patients in the Japanese study). However, our data show the same seasonal pattern for SAT in the year 2020 with hardly any cases of *Enterovirus* infection due to hygienic and behavioral measures introduced during the COVID-19 pandemic. Moreover, our data do not show any positive correlation with other viruses. The observed negative correlation with influenza viruses obviously cannot be interpreted as a protective effect of influenza viruses, but merely reflects the typical seasonal distribution in temperate zones with the highest numbers during colder months of the year (37). Due to the changes in the framework conditions during the pandemic and the high-quality surveillance data for respiratory infections and their causative agents, our data provide a high level of evidence and clearly argue against respiratory viruses, especially enteroviruses, as the cause of SAT.

Some other infectious diseases suspected of triggering SAT, such as measles and rubella, have become increasingly rare in Germany. The incidence of measles and rubella also reached a historic low in Germany in 2020. Both viruses have no specific seasonal pattern, but occur in sporadic outbreaks (16). In the years 2015 to 2019, measles was negatively correlated with SAT, which corresponds with the worldwide seasonality of measles (38). For 2020, the negative correlation was not statistically significant.

In the case of hepatitis C and HIV, it is often difficult to determine the exact time of infection. Interestingly, HIV showed a negative correlation with SAT incidence, but it may be assumed that SAT occurring several years after the infection is not causally related, especially since a protective effect of HIV is not plausible. In cases where SAT had been associated with hepatitis C, immunomodulatory therapy with interferon-α was discussed as a trigger rather than the infection itself (9). A statistical correlation could not be shown.

During the years 2015 to 2019, HEV incidence was positively correlated with SAT. The previously observed seasonality of HEV has been discussed as being related to leisure activities and dietary behavior in the summer months (39). As with enteroviruses, this correlation was disrupted in 2020.

No valid epidemiological data for EBV were available. But notably, infections peak in the first decade of life and therefore do not coincide with the SAT peak in the fifth decade of life (40).

Whether these infections may still have been triggering factors for SAT in some cases remains an open question. Coincidence, however, appears more plausible than causality. The question therefore remains as to what else could be the cause of the apparent seasonal distribution of SAT with the highest incidence during the summer months. Seasonality has been described for many autoimmune diseases with peak times differing among various diseases. Besides seasonality of infections, several factors have been used as explanations, including vitamin D and melatonin levels and UV radiation (41). The influence of ambient temperature on immunity has been demonstrated previously and is a growing concern as climate change progresses (42). Though speculative, it is therefore conceivable that the higher temperatures themselves are responsible for the increased incidence in summer.

In the years of the COVID-19 pandemic, it has been hypothesized that SARS-CoV-2 may have played a causative role in the development of SAT. The supporting data are rather inconclusive, and various opposing theories exist regarding specific clinical patterns of SAT following COVID-19 or the interpretation of a possible causal relation (9). While the previously mentioned Korean study indicates a slight increase in SAT cases during the SARS-CoV-2 pandemic (18), the Destatis data used in our study show no statistically significant difference. In 2020 in particular, the number of COVID-19 cases in Germany was well documented at fewer than 2 million thanks to consistent testing strategies (43). The vast majority of SARS-CoV-2 infections occurred after 2020, with a peak incidence in March 2022 (44). The seasonal distribution of COVID-19 does not match the seasonal distribution of SAT, which was unaltered throughout the pandemic years 2020 to 2022. On an epidemiological level, this observation provides strong evidence against a relevant influence of SARS-CoV-2 on the etiology of SAT. This is also supported by the study by Batman et al (24), who report that there is no clinical difference between SAT occurring in temporal correlation to COVID-19, SARS-CoV-2 vaccination, or neither. In recent years many cases and case series have been published on the temporal correlation between COVID-19 and SAT (9). The resulting perception of a causative correlation may be driven by a general tendency to publish COVID-19–related science (45). According to our results, however, this correlation appears to be coincidental.

### Conclusions

We have confirmed the previously observed seasonality of SAT. However, we show that the seasonality was not affected by the SARS-CoV-2 pandemic and the resulting infection prevention measures with their massive influence on the epidemiology of infectious diseases.

We thus provide strong evidence against previous suggestions of enterovirus infections as a potential cause of SAT, and against a correlation of SAT with other viruses, including SARS-CoV-2.

### Limitations

The Destatis data depend on ICD-10 coding from hospitals. As Destatis provides only statistical data, but—due to data protection issues—no access to individual patient data, it is methodologically not possible to verify the individual diagnoses. The remaining uncertainty regarding the correctness of diagnoses represents the greatest limitation of our study. However, since the health insurance companies frequently check the invoices, and errors are penalized financially, the motivation to submit correct data is high. The validity of diagnostic codes such as ICD-10 has been evaluated for different diseases, and a high degree of reliability has been demonstrated for diagnoses that are not based on complex definitions (46). The clinical and laboratory features of SAT usually allow a clear diagnosis, and although this has never been specifically investigated, a high level of reliability can be assumed here. Misdiagnoses and incorrect coding are still possible, but a major bias is unlikely because the diagnoses of all included periods would be affected in the same way.

Furthermore, the registry data include only hospitalized patients while SAT usually does not require in-hospital treatment. A behavioral change with regard to visiting medical providers was reported during the COVID-19 pandemic, which may have influenced the number of hospital admissions (47). This is reflected by the total number of hospital admissions from the Destatis data, but may have had a disproportionate effect on diseases such as SAT that are not life-threatening. On the other hand, in the early stages of the pandemic, treatment of patients with suspected viral disease (including SAT) was sometimes refused by private practices. These patients may therefore have more frequently visited hospitals. Whether and to what extent these pandemic-related effects on the German health-care system influenced hospital admissions due to SAT cannot be conclusively determined.

Ultimately, it can be assumed that only a minority of all patients suffering from SAT during the study period are included, which may have reduced statistical accuracy. The exact incidence of SAT in Germany is unknown. When assuming an incidence of SAT of 4.9 per 100 000/year as described for Olmsted County, Minnesota, by Fatourechi et al (1), the expected number for Germany with approximately 82.3 million inhabitants would be 36 294 for the period from 2016 to 2022. The 6856 hospitalized patients included in our study would therefore represent 19% of the expected cases. The data show a typical sex distribution (72% female and 28% male) and age distribution (>50% of cases during the fifth decade) as previously described in the literature (5), and can thus be considered largely representative of all SAT cases in the years analyzed.

On the other hand, relevant epidemiological studies on SAT are often limited because of single-center data and small cohort sizes (1, 2, 11, 12). In particular, the study by Martino et al (12), on which the assumption of a causal correlation between SAT and echovirus and coxsackievirus is based, contains only 80 cases. We therefore assume that, despite the aforementioned limitations, our study makes a valuable contribution to the ongoing discussion on the etiology of SAT.

By summarizing the data pertaining to years, the SAT fractions and pathogen infection rates are much more robust, but this also limits the data size to 12 points per year. This introduces uncertainty that cannot be resolved unless a more comprehensive, for example, Europe-wide survey, allows a resolution at the weekly level.

## Data Availability

All underlying data are publicly available from the listed sources.

## Abbreviations

CMV: cytomegalovirus
EBV: Epstein-Barr virus
HCoV: human coronavirus
HEV: hepatitis E virus
HLA: human leukocyte antigens
ICD-10: International Classification of Diseases, Tenth Revision
MSE: mean squared error
RKI: Robert Koch Institute
RSV: respiratory syncytial virus
SAT: subacute thyroiditis
